# Conservation analysis of sequences flanking the testis-determining gene *Sry* in 17 mammalian species

**DOI:** 10.1186/s12861-015-0085-6

**Published:** 2015-10-06

**Authors:** Christian Larney, Timothy L. Bailey, Peter Koopman

**Affiliations:** Institute for Molecular Bioscience, The University of Queensland, Brisbane, QLD, 4072 Australia

**Keywords:** *SRY*, Sex determination, Y chromosome, Gene regulation, Testis, Gonad

## Abstract

**Background:**

Sex determination in mammals requires expression of the Y-linked gene *Sry* in the bipotential genital ridges of the XY embryo. Even minor delay of the onset of *Sry* expression can result in XY sex reversal, highlighting the need for accurate gene regulation during sex determination. However, the location of critical regulatory elements remains unknown. Here, we analysed *Sry* flanking sequences across many species, using newly available genome sequences and computational tools, to better understand *Sry*’s genomic context and to identify conserved regions predictive of functional roles.

**Methods:**

Flanking sequences from 17 species were analysed using both global and local sequence alignment methods. Multiple motif searches were employed to characterise common motifs in otherwise unconserved sequence.

**Results:**

We identified position-specific conservation of binding motifs for multiple transcription factor families, including GATA binding factors and Oct/Sox dimers. In contrast with the landscape of extremely low sequence conservation around the *Sry* coding region, our analysis highlighted a strongly conserved interval of ~106 bp within the *Sry* promoter (which we term the *Sry* Proximal Conserved Interval, SPCI). We further report that inverted repeats flanking murine *Sry* are much larger than previously recognised.

**Conclusions:**

The unusually fast pace of sequence drift on the Y chromosome sharpens the likely functional significance of both the SPCI and the identified binding motifs, providing a basis for future studies of the role(s) of these elements in *Sry* regulation.

**Electronic supplementary material:**

The online version of this article (doi:10.1186/s12861-015-0085-6) contains supplementary material, which is available to authorized users.

## Background

Expression of *Sry*, a gene located on the Y chromosome, is required for differentiation of mammalian bipotential genital ridges into testes, a role evinced by the development of testes in XX mice with a 14.6 kb transgenic construct containing *Sry* and no other genes [1]. SRY initiates testis development by binding to a testis-specific enhancer of *Sox9* [2], a gene with a highly conserved role at the centre of the testis development program.

Perhaps surprisingly for a gene with such profound developmental consequences, *Sry* expression is required only in a small population of cells of the developing genital ridges to initiate male development [3]. In mice, initial expression of *Sry* at 10.5 days post coitum (dpc) is restricted to the central region of the genital ridge, but expands to fill the entire gonad by 11.5 dpc, before being extinguished to undetectable levels by 12.5 dpc [4, 5]. This short window of expression is so barely adequate for the task that delays of just a few hours lead to either ovarian or ovotestis development [6]. If *SRY* regulation is similarly critical in humans, it seems likely that improved understanding of factors and pathways regulating *Sry* will explain some undiagnosed XY disorders of sex development.

Previous studies have implicated a variety of factors in regulating *Sry* (reviewed in [7]) but specific *cis*-regulatory sites for these factors remain obscure. Assays such as ChIP-seq, typically used to identify regulatory elements, founder on a paucity of suitable tissue, as the small number of cells in which *Sry* is expressed render *in vivo* tissue collection difficult, and known *Sry*-expressing cell lines such as NT2-D1 and HepG2 do not recapitulate the expression profile of gonadal cells where *Sry* is expressed. These difficulties have led to a number of attempts to identify *cis*-regulatory regions *in silico*, by locating conserved regions in aligned *Sry* 5’ flanking sequences [8–12]. While contemporary tools make this an easy task for most genes, a lack of informative Y chromosome sequence has continued to hamper similar studies for Y-linked genes such as *Sry*. The lack of Y chromosome sequence can be traced to a strong bias towards using female samples in sequencing projects [13], a situation being addressed by the Y Chromosome Genome Project (https://www.hgsc.bcm.edu/y-chromosome-genome-project). Meanwhile, reports of *in silico* analysis of *Sry* flanking regions have been restricted to sequences from relatively few species, and at most a few kilobases in length.

Here, we present an analysis of sequences an order of magnitude longer than has previously been possible, and from a broader range of species. In addition to both global and local sequence alignment methods, we use several different sequence motif analyses and demonstrate that flanking regions of *Sry* vary considerably, even between closely related species, boosting the likelihood that conserved regions and motifs in the proximal promoter are indeed functional. Building on this result, we also hypothesise that proximal elements are the only regulatory sites required for *Sry*’s conserved testis-determining role.

## Results

### Locating Sry flanking sequences

The most recent published study of *Sry* flanking sequences [11] analysed some 8 kb of 5’ flanking sequence from five species. With the availability of additional genome sequence resources, we had the opportunity to identify both 5’ and 3’ flanking sequences in 18 different species, ranging in length from 2.3 kb to several megabases. Sources for these data included four whole genomes with complete or partial Y chromosome sequence, a published whole Y chromosome not part of a standard build [14], and *Sry*-containing sequences from Genbank for an additional 13 species with between 3.5 and 454 kb of 5’ flanking sequence, and between 2.3 and 470 kb of 3’ flanking sequence (Table 1).

**Table 1:** Sequences containing Sry and its flanking regions

Species	Source	Sense	Start	End	5’	3’
Antelope [52]	Genbank:NW_005810830	+	454 385	455 107	454 kb	470 kb
Bat [53]	Genbank:NW_005357697	+	131 171	131 827	131 kb	3.5 kb
Chimpanzee [54]	panTro3:chrY	+	23 812 571	23 813 185		
Cow [55]	bosTau7:chrY	-	42 225 210	42 225 899		
Ferret	Genbank:NW_004577527	+	1 715	2 374	1.7 kb	250 kb
Goat [11]	Genbank:EU581862	+	3 458	4 180	3.5 kb	2.3 kb
Horse	Genbank:AC215855	+	166 197	166 904	166 kb	12 kb
Human [56]	hg19:chrY	-	2 655 030	2 655 644		
Macaque [14]	Supplementary Data 1	-	81 625	82 236	11 Mb	81 kb
Marmoset	Genbank:AC221052	-	165 854	166 537	50 kb	165 kb
Mouse [57]	mm9:chrY	-	1 918 381	1 919 568		
Pig [58]	Genbank:NW_003612981Genbank:NW_003536874	+, −	33 265, 122 106	33 975, 122 816	33 kb, 20 kb	70 kb, 122 kb
Rabbit [59]	Genbank:HM230423^a^	-, +	3 435, 61 035	4 058, 61 658	90 kb, 60 kb	3 kb, 30 kb
Sheep [60]	Gebank:Z30265^b^, Genbank:AF026566^c^	+, +	1	723	4.8 kb	
Tiger [61]	Genbank:NW_006712469	+	8 618	9 322	8.6 kb	13 kb
Walrus	Genbank:NW_004451818	-	15 244	15 906	110 kb	16 kb
Whale [62]	Genbank:NW_006729534	-	97 353	97 967	11 kb	97 kb

Genomic sequences used in this study. Sense indicates the strand where *Sry* is located in each species, with Start and End indicating the position of the *Sry* coding region within the sequence. 5’ and 3’ provide an estimate of how much flanking sequence is available in each direction. Sequences listed without citation are unpublished

^a^HM230423 contains two copies of *Sry*

^b^Z30265 contains the sheep CDS only

^c^AF026566 contains sheep 5’ flanking sequence only

Despite locating flanking sequences from 18 species, only 17 were used in our analysis. Sequences from the rat were excluded on the basis of high copy number [15], and inability to determine which copy(/ies) may be testis-determining. Though the rat has the highest *Sry* copy number (11) among the species we examined, duplicate copies of *Sry* are also known to be present in pig and rabbit. In those species, comparison of the copies revealed coding and flanking sequences to be virtually indistinguishable. For this reason, we arbitrarily chose a single copy from each species to include in the analysis.

### Global alignment of open reading frames

**Fig. 1:**
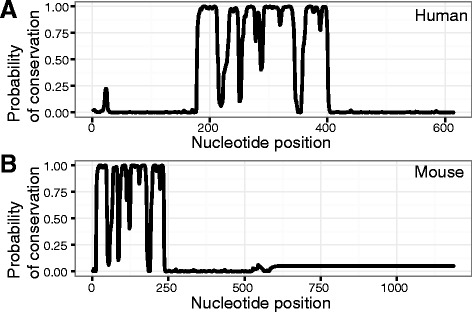
Conservation in *Sry* coding sequences is restricted to the HMG-box. **a** and **b** depict the probability of conservation of individual bases within the *Sry* ORF (defined in Table 1), as measured by RPhast, based on a multiple alignment by MUSCLE of the *Sry* coding regions from 17 species. The reference sequence for **a** is from human, while the reference sequence for **b** comes from mouse. In both panels, regions of high conservation correspond to known locations of human and mouse HMG boxes

As not all sequences included in this analysis have been functionally confirmed as testis-determining loci, we began by verifying expected conservation of the *Sry* coding region, reasoning that this would also indicate functional conservation. Coding sequences from 17 species (Additional file 1) were globally aligned with MUSCLE [16] and conservation quantified with RPhast [17], confirming that little sequence similarity exists outside the HMG box (Fig. 1). The HMG box was in the central position in all species except the mouse. Conservation of the HMG box in all cases led us to conclude that all sequences represent testis-determining loci.

### Global alignment of flanking sequences

**Fig. 2:**
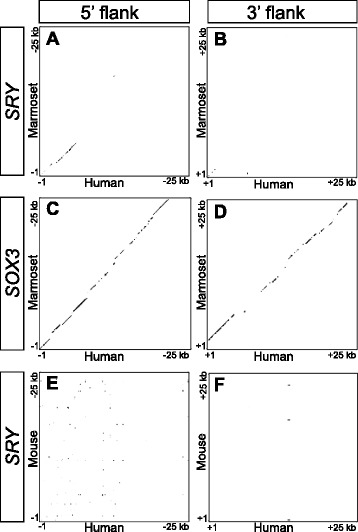
Flanking sequences of *Sry* are less conserved than those of Sox3. A series of dotplot comparisons between 25 kb sequences flanking orthologous genes in human and another species. **a** The 5’ flank of *SRY* between human and marmoset. **b** The 3’ flank of *SRY* between human and marmoset. **c** The 5’ flank of SOX3 between human and marmoset. **d** The 3’ flank of SOX3 between human and marmoset. **e** The 5’ flank of *SRY* between human and mouse. **f** The 3’ flank of *SRY* between human and mouse. All dot plots were generated by GEPARD (see Methods for details)

We next visualised the level of gross conservation in the flanks of *Sry*. Dot plots revealed considerable divergence in the 25 kb of sequence immediately flanking the 5’ and 3’ ends of *Sry*, even between closely related species such as humans and marmosets (Fig. 2a, b), two species with a last common ancestor only 35–40 million years ago. Conservation was restricted to the proximal 5 kb of the 5’ flank (Fig. 2a), and was totally absent in the 3’ flank (Fig. 2b). In contrast, a comparison between the same two species for *Sox3*, an X-linked gene thought to share a common ancestor with *Sry* [18], revealed far greater similarity (Fig. 2c, d). A similarly constructed dot plot comparison of human and mouse *Sry* flanking sequences (Fig. 2e, f) revealed virtually no observable conservation in the 25 kb region upstream of the *Sry* ATG. We conclude that the DNA sequence flanking *Sry* has been subject to a much greater mutation rate than its counterpart in *Sox3*.

**Fig. 3:**
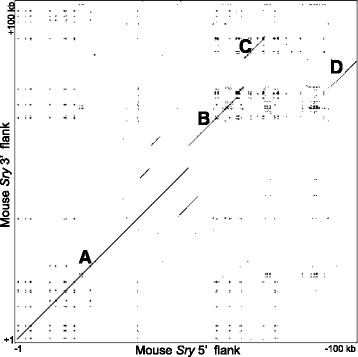
Murine *Sry* is flanked by inverted repeats extending for more than 50 kb. Figure shows a GEPARD dotplot comparing 100 kb of the murine *Sry* 5’ and 3’ flanks. Conserved intervals are shown as **a**-**d**

Previous reports of the open reading frame (ORF) of mouse *Sry* have placed it within just 2.8 kb of unique DNA surrounded by inverted repeats at least 15.5 kb in size [19]. To resolve the question of how long these repeat regions are, we also generated a dot plot between the 5’ and 3’ flanking regions of *Sry* in the mouse (Fig. 3), and observed that the inverted repeats extend unbroken for some 50 kb (Fig. 3a). Other extended regions of identity were observed between the 5’ and 3’ flanks as much as 100 kb distal to the ORF (Fig. 3b-d), suggesting the original duplication event was of at least this size, and that a series of smaller insertions and deletions have occurred since. A chromosomal rearrangement within one arm of the repeat has occurred, such that the positions of regions C and D are reversed between the 5’ and 3’ flanks (Fig. 3c, d). The paucity of secondary (off diagonal) lines in the figure indicates little internal structure within the inverted repeats.

**Fig. 4:**
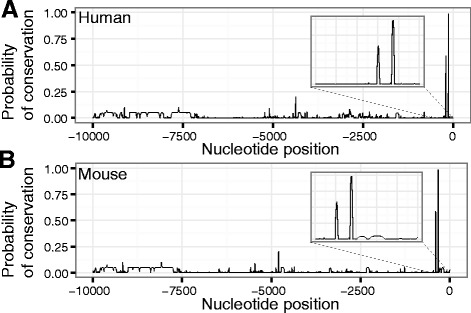
Just two short conserved regions are present in the 10 kb 5’ of *Sry* in human and mouse. Plots of the probability of conservation in the 10 kb region upstream of the start of translation of the *Sry* gene in (**a**) human and (**b**) mouse. Conservation is estimated by RPhast from a multiple alignment generated by MUSCLE using repeat-masked flanking sequences of 17 species

To quantify the level of conservation in the flanking regions, we took 10 kb sequences from immediately 5’ of the *Sry* start codon in 17 species, masked known repeats using RepeatMasker [20], and then aligned the sequences using MUSCLE [16] (Additional file 2). Estimating the probability of conservation at each position of the resulting alignment with RPhast [17] (Fig. 4), we observed in both human and mouse two closely spaced conserved regions just a few hundred base pairs 5’ of the ORF (Fig. 4, insets). A similar analysis of 10 kb flanking sequences from 3’ of the gene (Additional file 3) found only two short, incompletely conserved regions (results not shown).

**Fig. 5:**
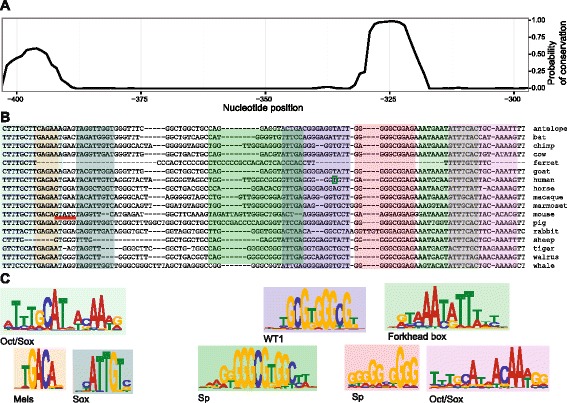
The highly conserved SPCI contains recognised TF binding motifs. **a** A detailed plot of conservation in the SPCI, covering bases from −402 to −296 in the 5’ flank of mouse *Sry*. Conservation is estimated by RPhast from a multiple alignment generated by MUSCLE using repeat-masked flanking sequences of 17 species. This plot is aligned with (**b**) the sequence of the multiple alignment from which the conservation probabilities were derived. The human TSS is indicated by a small green box (TSS in other species is largely uncharacterised). A red underline indicates a putative GATA4 binding site present in the mouse whose function is supported by *in vivo* evidence. **c** A representative selection of logos for motifs with binding sites predicted by FIMO within the sequence. Details for motifs, and *p*-values calculated by FIMO are, from left to right: Oct/Sox (JASPAR MA0142.1, *p*-value < 10^−4^), Meis (Jolma MEIS1_DBD, *p*-value < 10^−4^), Sox (JASPAR MA0078.1, *p*-value < 10^−4^), Sp (JASPAR MA0079.1, *p*-value < 10^−5^), WT1 (Jolma EGR1_DBD, *p*-value < 10^−4^), Sp (JASPAR MA0079.2, *p*-value < 10^−4^), Forkhead box (Jolma FOXD2_DBD, *p*-value < 10^−5^), Oct/Sox (JASPAR MA0143.1, *p*-value < 10^−4^)

A more detailed analysis of the interval spanning the two conserved regions (which we designate the *Sry* proximal conserved interval, or SPCI; Fig. 5a, b; Additional file 4) showed that it covered 106 bp of the human sequence (hg19 - chrY:2655744–2655849, position −204 to −99 relative to the translation start site [XSS]) overlapping the transcription start site (TSS). The corresponding mouse sequence (mm9 – chrY:1919865–1919971, XSS-relative position −402 to −296) is 107 bp in length, and has its proximal end 25 bp 5’ of the TSS.

### Motif scanning and enrichment

Having identified the SPCI as the most conserved part of the *Sry* flanking sequence, we subsequently wanted to locate potential transcription factor (TF) binding sites. To this end we first scanned the SPCI sequence of human and mouse (Additional file 4) using FIMO [21] and 1270 motifs from three major databases (see Methods). The strongest result, at the 5’ limit of the sequence, was to a previously unreported instance of an Oct/Sox motif (MA0142.1 from the JASPAR database; *p*-value = 3x10^−5^; Fig. 5c). Other previously unreported potential binding sites, including those for Meis and Forkhead factors, were also present (Fig. 5c), along with a reported WT1 motif [22] (EGR1_DBD from the Jolma database; *p*-value = 3x10^−5^), coincident with the human TSS.

We next simultaneously scanned multiple sequences for motifs with CentriMo [23]. CentriMo searches for motif occurrences at similar positions in multiple sequences, so it was first necessary to re-align the ungapped sequences using a position other than the XSS. We chose the base that RPhast had identified as the most conserved (Fig. 5a), and determined the position of this base in each sequence (Additional file 5: Table S2). Using these positions as references, we then took the sequences extending for 100 bases in the 5’ direction and 500 bases in the 3’ direction (for a total of 600 bp; Additional file 6), and scanned them for 1270 motifs, from the same databases mentioned previously, using CentriMo (Additional file 7).

**Fig. 6:**
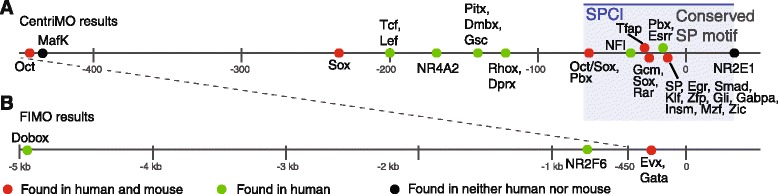
TF binding motifs are locally enriched in non-conserved *Sry* flanking sequences. Motifs are positioned at the centres of the windows in which they were found, and are colour-coded to indicate presence in mouse and/or human. The origin (labelled as 0) is 7 bp downstream of the annotated human TSS (hg19), and corresponds to a conserved SP factor motif. Additional file 5: Table S2 lists the positions of the origin in other species. Motifs were included in this figure only if they were detected in at least eight species, and their presence had an E-value no greater than 1 (with two exceptions, see below). **a** Sites found by CentriMo in 600 bp sequences from 17 species (Additional file 7, wherein position +200 corresponds to the figure origin; Additional file 8), with the SPCI indicated by a shaded blue box. NR4A2 is included for biological relevance, despite an E-value of 6. **b** Additional sites detected by FIMO in corresponding 100 bp windows of 10 kb sequences from 13 species (Additional file 10). NR2F6 is included for biological relevance, despite its presence in only seven species

The results showed a number of windows in the sequences that contained the same motif in multiple species, most of which were concentrated near the original alignment point (Fig. 6a, Table 2, Additional file 8). Motifs found to be enriched are able to bind known regulators including WT1 and NR5A1, as well as novel putative regulators, including a range of Oct and Sox family members. The significance levels attributed to these results should be considered in the context of the high levels of redundancy among the motifs we used in our comparison. This redundancy is due both to multiple databases containing entries for the same factors, and also to the propensity for related factors to bind to the same or similar motifs. Consequently, the multiple-testing correction afforded by the E-value statistic is likely a highly conservative estimate of the true significance of these motif occurrences.

**Table 2:** Enriched motifs identified by CentriMo and FIMO

Figure 6 Label	Motif DB	Motif ID	E-value	Position relative to anchor SP motif
Panel A				
Oct	Jolma	POU3F4_DBD	2x10^−6^	−443
MafK	Jolma	MAFK_full	0.5	−435
Sox	JASPAR	MA087.1	10^−3^	−234
Tcf, Lef	UniPROBE	UP00054_1	5x10^−4^	−197
NR4A2	JASPAR	MA0160.1	6	−169
Pitx, Dmbx, Gsc	Jolma	PITX3_DBD	0.3	−142
Rhox, Dprx	Jolma	RHOXF1_DBD_2	0.03	−127
Oct/Sox, Pbx	JASPAR	MA0142.1	10^−6^	−66
NFI	JASPAR	MA0119.1	3x10^−6^	−36
Tfap	Jolma	TFAP2C_full	3x10^−6^	−30
Gcm, Sox, Rar	UniPROBE	UP00048_2	3x10^−3^	−24
Pbx, Esrr	UniPROBE	UP00079_2	0.8	−18
SP, Egr, Smad, Klf, Zfp, Gli, Gabpa, Insm, Mzf, Zic	UniPROBE	UP00007_2	3x10^−10^	−12
NR2E1	Jolma	NR2E1_full	0.4	+33
Panel B				
Dobox	UniPROBE	UP00232_1	1	−4976
NR2F6	Jolma	NR2F6_DBD_2	0.03	−760
Evx, Gata	Jolma	EVX1_DBD	0.01	−293

Details of the motifs depicted in Fig. 6. The first column corresponds to the labels used, from left to right, in Fig. 6. The next two columns refer to the specific motif id, and its containing database, that provides the smallest E-value for motifs at or around that particular site within the sequence (subject to the additional constraint that included motifs must also be present in at least 8 species). The final column reports an E-value (to 1 significant figure) for each of the motifs. Although this table reports a single motif at each site, redundancy of databases and similarity of binding sites for multiple transcription factors means that multiple motifs are typically found to be enriched at each of these sites. Full results can be found in Additional file 8 for Panel A, and Additional file 10 for Panel B

### Extended analysis of locally enriched motifs

CentriMo’s simultaneous consideration of multiple sequences allows it to incorporate information about conservation that is unavailable to a single-sequence scanning tool like FIMO. CentriMo, however, considers only the highest-ranked match for a motif in each sequence. This constraint is unrestrictive with the large numbers of short sequences on which CentriMo is typically used. On the small number of long sequences in our analysis, however, it is far more likely to overlook conserved motifs. To counter this possibility, we next developed a method to combine FIMO’s consideration of all matches in a sequence with CentriMo’s simultaneity.

Using the reference nucleotide from the global multiple alignment, as described previously, we generated two additional sets of unmasked flanking sequences. The first set contained 1 kb sequences (100 bp 5’ of the reference base, and 900 bp 3’) from 17 species, while the second set consisted of 10 kb sequences (100 bp 5’ of the reference base, and 9.9 kb 3’) from 13 species (insufficient sequence was available for the ferret, goat, sheep, and tiger).

We independently analysed each sequence with FIMO, and processed the results to determine positions where a statistically unlikely number of sequences (see Methods) contained a match for the same motif within either disjoint or 50 % overlapping windows of 100 bp or 250 bp. This approach greatly lowered the burden of multiple tests compared to CentriMo, which considers all possible window sizes at all possible positions within tendered sequences. This approach to locating motifs differs from typical alignment-based approaches in two important ways. Firstly, it is targeted directly at conservation of motifs, not necessarily of the underlying sequence. Divergent sequences that retain the ability to bind a particular transcription factor will be captured by this method where they might be overlooked by sequence alignment. Secondly, the use of windows allows us to capture binding motif occurrences that have drifted as species diverged. Whereas sequence alignment approaches require a motif to be at the same position in multiple sequences, our approach requires only that a motif be within the same 100/250 bp window between different species.

Within the region previously analysed with CentriMo, this method found largely the same likely motifs at the same positions (Additional file 9). One result, however, both unreported and undetected by our earlier analyses, was a GATA-like motif 260 bp 3’ of the reference nucleotide that was present in human, mouse, and six additional species (Fig. 6b, Additional file 10). GATA4 is an essential factor in testis determination [22, 24, 25], but precise *in vivo* binding sites remain uncharacterised. This result provides a putative site suitable for further functional analysis. Several additional motif occurrences were also found at more distant positions (Fig. 6b, Additional file 10), but, unlike the more proximal motifs, were present only in 4–6 species (or fewer than half of the 13 available). None of these distal motifs was present in the mouse.

### De novo motif discovery

Having exhaustively scanned for known motifs, we also wanted to know if the flanks of *Sry* contain conserved *de novo* motifs. We found the high dimensionality and small sample size of our data rendered PWM-based motif discovery tools (eg. MEME) ineffective, so we instead turned to the string-based WeederH program [26]. This program places even fewer restrictions on the search for motifs than our previous combination of FIMO and sequence windows. Whereas that analysis required known motifs to be located at similar positions in multiple sequences, WeederH searches for similar sequences of nucleotides at any positions in the input sequences, regardless of whether or not they represent a known motif. We compared results from 10 kb unmasked sequences from nine species with control results from shuffled sequences (see Methods) to generate a set of background scores and a false discovery rate. This method found four motifs to be significant at a false discovery rate of 10 % with human as the reference, with the two most significant results being found in the SPCI (Table 3). A further two motifs were found to be significant when mouse was used as the reference. There was no overlap between the results using the two different reference sequences.

**Table 3:** *De novo* motifs in *Sry* flanking sequences have potential to bind known transcription factors

Start Position	End Position	Sequence	Score	q-value (conservation)	Database of best match	Top-ranked motif ID (based on q-value)	Factor binding motif	q-value (motif scan)
Human Ref								
−132	−121	GGGCGGAGAAAT	13.71	0.01	JASPAR	MA0079.2	SP1	9x10^−3^
−204	−193	TTTGCTTGAGAA	10.26	0.02	JASPAR	MA0142.1	POU5F1	3x10^−3^
−1 526	−1 515	TTTTCAAGGTTC	9.44	0.02	JASPAR	MA0017.1	NR2F1	1x10^−3^
−5 152	−5 141	AAAGTGACCTTC	7.80	0.08	Jolma	ESRRG_full_3	ESRRG	1x10^−3^
Mouse Ref								
−467	−456	GAAAAAGCGATA	12.16	0.01	Jolma	ONECUT2_DBD	ONECUT2	6x10^−3^
−425	−418	TAACATTC	9.47	0.05	Jolma	HSF1_full	HSF1	3x10^−2^

Conserved sequences identified by WeederH (q-value < 0.1) using either human or mouse as a reference. Significance of scores provided by WeederH was assessed by comparing actual scores to scores obtained from 100 random shufflings of the non-reference sequences (see Methods). Sequences shown here were extended by an additional five nucleotides on both ends from the relevant genomic position before being scanned with FIMO. The top-ranking motif for each sequence is reported here regardless of the q-value reported by FIMO. POUF51 is also known as OCT4. NR2F1 is also known as COUP-TF1

To establish the novelty of motifs reported by WeederH, we extended the WeederH-predicted sites with the five base pairs adjoining them at each end in the genomic sequence of the reference species, and scanned these extended motifs with FIMO (Table 3). FIMO found significant matches for all four motifs identified using the human reference. The first two motifs (both located in the SPCI) exhibited greater similarity to Sp1 and Oct/Sox binding sites, respectively, than to any other known transcription factor binding motifs, recapitulating earlier results. The remaining two motifs were found to best match the motif for NR2F1 (also known as COUP-TF1; MA0017.1 from the JASPAR database; q-value = 10^−3^) and an estrogen-related receptor motif (ESRRG_full_3 from the Jolma database, q-value = 10^−3^). The estrogen-related receptor motif provides a possible binding site for the known *Sry* regulator NR5A1, while the putative match for NR2F1 is interesting in light of the possible role of the related NR2F2 (also known as COUP-TFII) in gonad development [27].

The two motifs found using the mouse as a reference also had significant similarity with known motifs. The first was found to best match a motif for the onecut family of transcription factors (ONECUT2_DBD from the Jolma database; q-value = 6 × 10^−3^) (Table 3). Onecut factors play roles in C. elegans sex determination [28], but have not previously been implicated in the corresponding mammalian process. The second showed best agreement with a heat-shock motif (HSF1_full from the Jolma database; q-value = 3 × 10^−2^). Heat-shock proteins are known to play roles in spermatogenesis [29] in mice, and have also been found to be enriched in the testis of the swamp eel [30], but have no known role in sex determination.

### Pairwise local alignment

Given the limited degree of conservation observed in the 10 kb adjacent to the XSS, we reasoned that conserved regulatory elements might instead lie in more distal positions. We first attempted to globally align longer flanking sequences, but observed that the majority of alignment tools were confounded by them, resulting in a variety of error conditions. With this obstacle in mind, and also taking into consideration the potential for rearrangement of the Y chromosome, we decided to instead search more distal positions for occurrences of local similarity. Using repeat-masked 100 kb intervals from the 5’ and 3’ flanks of *Sry*, we generated a series of pairwise local alignments between the human sequence and the corresponding sequence from each of five other species where extended flanking sequence was available.

**Fig. 7:**
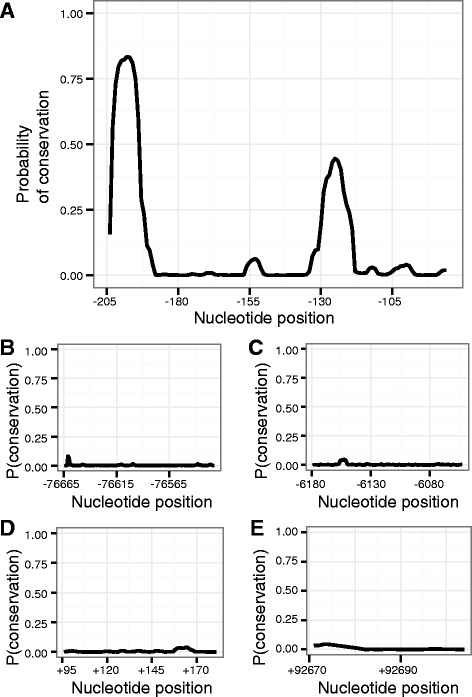
No further sequence conservation is detected in alignments of short flanking regions up to 100 kb distal to *Sry*. Each panel plots the probability of conservation in a multiple alignment of short sequences previously predicted by LALIGN to share pairwise similarity with the same stretch of the human sequence. Multiple alignments are generated by MUSCLE, with conservation estimated by RPhast. Values on the x-axis indicate the position relative to the human *Sry* ORF, with negative values 5’ of the gene, and positive values 3’ to the gene. **a** The SPCI is successfully rediscovered by this method, with the same two peaks of conservation as seen in Fig. 5. **b, c** Two representative results from the 5’ flank showing the low probability of broad conservation seen in all results other than (**a**). **d**, **e** Two representative results from the 3’ flank showing the low probability of broad conservation seen in all 3’ results

The results from this preliminary analysis were, for each of the five alignments, a list of all the paired regions from the flanks of *Sry* that LALIGN reported as similar between the human sequence and the sequence of one of the other species under consideration. To evaluate how widespread this similarity might be, we next compared the sets of paired regions resulting from each of the five local alignments. We were interested in locating those parts of the human sequence found similar to a part of the flanking sequence of every one of the other species it had been aligned against. That is, we found the paired regions, across all comparisons, where the human halves of the pairs overlapped. Within each of these sets, we then took the other half of each pair, the sequences found similar to the sequence in humans, and globally aligned them (Fig. 7). Conservation was assessed as for the global alignments discussed previously. We found that this method successfully rediscovered the SPCI, but that broad conservation was absent in all other multiple alignments, supporting the notion that conserved regulatory elements do not exist beyond the promoter.

## Discussion

We have applied a range of contemporary computational tools to the task of identifying conserved elements in the genomic sequence flanking the mammalian testis-determining gene *Sry*. In doing so, we have analysed flanking sequences an order of magnitude longer, and from a more diverse range of mammals, than has previously been possible for this gene. We took advantage of newly available Y chromosome sequences, which have historically been difficult to obtain, restricting earlier studies to smaller datasets, both in terms of species and sequence length.

Our initial global alignment of multiple repeat-masked sequences predicted, in a 10 kb region flanking the 5’ end of the gene, just one broadly conserved region, which we have termed the SPCI. A similar global alignment of the region flanking the 3’ end of the gene predicted no conserved regions at all. A variety of motif scanning techniques predicted conserved transcription factor binding sites as far as 5 kb upstream, but subsequent efforts to identify conserved sequences using local alignment tools returned only the SPCI, and made no further predictions of broad conservation, even when we considered sequence as distant as 100 kb upstream and downstream from *Sry*.

### The Sry proximal conserved interval

Throughout our analyses, the SPCI has consistently been identified as the most conserved interval in either flank of *Sry*. The SPCI is approximately 106 bp long, overlaps the TSS in humans, and is adjacent to it in mice. Conservation of this region has been identified in multiple previous studies, including between humans and mice [8], and between multiple primates [12]. Our analysis was unable to locate additional conserved regions in *Sry*’s flanking regions, but has demonstrated that the SPCI is more widely conserved than previously understood, and is found in the flanking region of *Sry* in diverse mammalian species (Fig. 5b). In fact, of the 17 species included in our multiple sequence alignment, the only two species to lack the SPCI were the sheep, where it has previously been reported as absent [9], and the ferret. Thus, our data highlight the broad extent of conservation of the SPCI in a region of the genome otherwise devoid of significant conservation.

Conservation of sequence suggests a conservation of function. In this regard it may be significant that we identified conserved motifs for transcription factors known to be important in gonad development within the SPCI, lending support to the notion that it is required for transcriptional regulation of *Sry*. The strongest conservation peak corresponds to an Sp1 motif (Fig. 5a, positions −330 to −320). Sp1 has been shown capable of regulating *Sry in vitro* [31], and previous studies have considered conservation of this region in a narrower range of species [12]. Immediately 5’ to this is a somewhat conserved sequence that has previously been investigated *in vitro* in the context of WT1 binding, where it has been found essential for WT1-dependent activation of the mouse *Sry* promoter [22].

A second, highly conserved element in the SPCI (Fig. 5a, positions −403 to −390) has been noted previously as conserved between smaller groups of species [8, 12], but our results indicate it to be far more widely conserved than previously reported. We found this element to closely resemble the binding motif of an Oct/Sox dimer.

Curiously, though our interest in this element of the SPCI stems from its conservation, the only functional evidence associated with it is attributable to a unique pentanucleotide, GATAC, a consensus GATA binding site, which is present on the reverse strand in the mouse sequence but in no other species. In *in vitro* experiments, GATA4 is able to bind to this site and activate mouse *Sry* promoter constructs transfected into HeLa cells [22]. *In vivo* experiments also implicate these bases, as they represent the only likely GATA4 binding site between the primer pairs of a ChIP-PCR assay that showed strong GATA4 binding at 11.5 dpc [25].

### Locally enriched motifs

While the SPCI represented the only well conserved stretch of sequence in our results, we also found a number of transcription factor binding motifs locally enriched within narrow intervals of 100 bp or less. In the absence of broader conservation at the sequence level, this localised conservation of motifs may indicate functional elements common to multiple species. Perhaps the most interesting result in this regard is the presence of four separate positions within the 450 bp 5’ of the conserved Sp1 site where the DNA sequence is permissive in the majority of species for binding by Oct and/or Sox factors. Also of interest are two further sites, one for an estrogen-related receptor motif, and another, some 160 bp upstream of the human TSS, which our analysis identified as a motif for NR4A2. Both these motifs are similar to that of NR5A1 (also known as both SF-1 and Ad4BP), a known regulator of *Sry* [10, 32], suggesting possible binding sites for this factor (a specific NR5A1 motif was not identified by our analysis as it was not present in any of the available databases). As with the conserved sequences of the SPCI, it remains unclear what, if any, functional role these elements might play in *Sry* regulation.

### Sry may not require distal regulatory elements

Our results indicate that, even between closely related species, conservation in the flanks of *Sry* is restricted to just a few kilobases 5’ of the TSS, with no discernible, widespread conservation in the 3’ flank, or in more distant parts of the 5’ flank. Our analysis, which included all available data for this genomic locus, used a number of analyses in an attempt to discern even low levels of conservation that might be present across the various available species. In addition to global alignment, we constructed a series of local alignments in the hope that these might reveal short, highly similar sequences, even if they were not at a consistent distance from the ORF across species. Contrary to our expectations, we instead conclusively demonstrated an absence of broadly conserved sequences. The only portions of sequence we did not analyse were the repeat regions masked by RepeatMasker [20]. While there is increasing evidence at the genomic scale that repeat regions can harbour regulatory function [33, 34], it remains unclear how putative regulatory sites in repeat regions might be predicted in the context of a single gene such as *Sry*.

The position of murine *Sry* between inverted repeats [19] suggests that, in the mouse, the gene has been transposed to its current location at some point in the past from an entirely different position on the Y chromosome. Given this observation, we might speculate that, in the mouse at least, all sequence specific regulatory elements necessary for male sex determination lie not only within the 8 kb of L741, the construct first used to generate transgenic XX male mice [1], but also within the few hundred base pairs of unique non-coding sequence that lie between the arms of the repeat. Available evidence accords with this view, with all suspected binding sites for transcription factors regulating mouse *Sry* lying within this region [7].

Finally, it is worth noting the possibility that sequence-specific transcription factors are not the primary drivers of *Sry* regulation, and that other factors, such as DNA methylation [35, 36] and epigenetic modification [37, 38] play critical roles. Conservation-based approaches would provide little insight in clarifying how *Sry* is regulated in this case.

### *Mouse* Sry *as a model of human* SRY *regulation*

While we compared 17 mammalian *Sry* sequences in this study, our analysis was anchored in mouse as the species in which sex-determination is currently best understood. It is pertinent to ask to what extent the study of mouse *Sry* regulation is likely to improve our understanding of the corresponding process in other mammals, and especially humans. *Sry* is strongly upregulated in XY gonads of both species during early development of the testes, and a variety of experiments have shown the potential for human *SRY* regulatory and coding sequences of *Sry* to function in mice (reviewed in [7]), implicating common factors in its regulation and downstream effects (eg. [22, 39]). Functional dissection of mouse putative regulatory elements followed by mutation analysis in undiagnosed cases of human XY gonadal dysgenesis is required to experimentally validate the utility of mouse as an experimental model for studying *Sry* regulation.

## Conclusions

Several transcription factors are known to regulate *Sry* during sex determination, but specific binding sites for these (or any other) factors remain uncharacterised. Using sequences an order of magnitude longer than previously available, we have applied a range of computational analyses to the task of identifying conserved regulatory elements of *Sry*. These analyses highlight a short, well-conserved sequence, which we have dubbed the SPCI; and reveal the large differences that otherwise exist between the flanking regions of *Sry* in different species.

Our results suggest a new model whereby the testis-determining role of *Sry* depends solely on a combination of transcription factor binding to the SPCI and epigenetic regulatory mechanisms. Testing this model will require the targeting of specific transcription factor binding sites within the SPCI with a genome editing system such as CRISPR/Cas [40].

## Methods

### Sequence acquisition

DNA sequences (Table 1) were obtained from a variety of sources. Bioconductor BSgenome packages were used to obtain flanking sequences for human, chimpanzee, mouse, and cow, as these species all have whole genome builds with significant Y chromosome sequences, and annotations for positions of *Sry*. We obtained flanking sequences from a supplementary FASTA file of a complete published Y chromosome sequence in macaque [14]. The position of *Sry* within the chromosome was established by downloading the macaque *Sry* mRNA sequence from Genbank [Genbank:NM_001032836], and searching the chromosomal sequence for an exact match.

For other species, we first searched the Genbank CoreNucleotide database with the query string “sry”. This resulted in a selection of mRNA and RefSeq sequences for *Sry* coding and promoter regions. The coding regions from this initial set of sequences were then used as query sequences in BLASTN searches against available genomic databases (NCBI Genomes, High throughout genomic sequences). Where these searches located sequences with embedded coding regions, they were accepted as bona fide *Sry* sequences, downloaded, and manipulated with the Biostrings package to obtain flanking sequences.

### Sequence manipulation

DNA sequences were manipulated in R [41], using a variety of packages from Bioconductor [42]. Where relevant sequences for a species were embedded in a whole genome build, flanking sequences were obtained using the relevant BSgenome package (Table 1). For all other species, FASTA files were downloaded from Genbank, and subsequently manipulated using the Biostrings package. The translation start site (XSS), rather than the TSS, was defined as position +1 in the coordinates for all sequences because the TSS is uncharacterised in some species.

### Repeat masking

Sequences were masked for repetitive elements using RepeatMasker [20]. Default parameters were used except for the DNA source. A value for this parameter must be selected from a range of pre-defined species/orders. For each sequence, we selected the option most closely related to the species where the sequence originated (Additional file 5: Table S1).

### Global sequence alignment and conservation

Sets of repeat-masked sequences were globally aligned by MUSCLE [16] using default parameters. Alignments were viewed, and minor adjustments made manually, using MEGA6 [43]. We then used RPhast [17], an R package, to estimate conservation. The phyloFit function, with default parameters, was first used to estimate a neutral model from the alignment of *Sry* coding regions and an associated guide tree. This model was then used in conjunction with RPhast’s phastCons function to estimate conservation in the previously aligned flanking sequences.

### Motif scanning

Two separate programs from the MEME suite [44] were used to scan unmasked sequences for motif occurrences. Individual sequences were scanned using FIMO version 4.9.0 [21] (with default parameters unless otherwise specified), while simultaneous scanning of multiple sequences used CentriMo 4.10.0 [23] with the optional –local flag. This flag allows identification of enriched motifs at any position in the sequences. In all motif scanning experiments, sequences were scanned with 1270 motifs from the JASPAR [45], Jolma [46], and UniPROBE [47] databases.

The analysis of motif occurrences within positional windows used, for each set of input sequences, two separate invocations of FIMO. The first used default parameters, while the second used the parameter --thresh 5e-4, a tolerance slightly less stringent than the default value of 1 × 10^−4^, in order to make the search more comprehensive, at the risk of more false positives. Output from FIMO was subsequently processed in R. Results were divided, in two separate analyses, into 100 bp and 250 bp windows. For each of these window sizes, alternative analyses considered either disjoint windows or windows with a 50 % overlap (ie. 100 bp windows were overlapped by 50 bp, 250 bp windows were overlapped by 125 bp). In all cases, we counted the number of species in which each motif appeared in each window. We derived a p-value for each event by first defining the probability of a motif occurring at any single base pair in a single sequence as the maximum of the FIMO threshold parameter (either 1 × 10^−4^ or 5 × 10^−4^) and each of the empirically observed probabilities (observed occurrences divided by total available positions), and then modeling the probability of multiple matches in a window as a binomial distribution. False discovery rate was calculated using Bioconductor’s qvalue package. In summary, the windowing analysis determined the probability of a motif occurrence at an individual base pair in any single sequence, and then extrapolated this, by way of a binomial distribution, to the probability of a motif occurring in the same window in multiple species. Parameters varied in this analysis were the sequence lengths (1 kb or 10 kb), the FIMO tolerance (1 × 10^−4^ or 5 × 10^−4^), the window size (100 bp or 250 bp), and whether or not the windows overlapped (true or false). Results are provided for each of the sixteen possible permutations of these parameters (Additional files 9 and 10).

### Motif discovery

*De novo* motif discovery used WeederH [26] on unmasked sequences, as the program accepts only A, C, G, and T as input. Different species, mouse and human, were used as reference species in two separate experiments. A negative control was established for each experiment by running WeederH with the reference sequence and 100 random shufflings of the nucleotides within each of the non-reference sequences. False discovery rate was established by comparing scores from actual observations to scores from the 100 trials with random shuffling. The sequence motifs reported by WeederH were compared to known motifs by first extending the reported motifs (either eight or twelve bp in length) by five base pairs on both sides using the endogenous context of the motif in the genome of the reference species — either human (hg19) or mouse (mm9). These extended strings were then analysed with FIMO using the parameter --thresh 5e-4.

### Local sequence alignment

Pairs of repeat-masked sequences were locally aligned with LALIGN [48] using default parameters. Output files from LALIGN were parsed and compared for overlapping local alignments using bespoke programs implemented in Racket [49] and R [41]. From each set of overlapping regions (which could potentially involve multiple disparate sequences from a single species), a single sequence was chosen from each species so as to maximize the overall length of the alignment. These sequences were then globally aligned with MUSCLE as described previously.

### Graphical output

Dot plots were generated by GEPARD [50], with command line options of –maxwidth 300 –maxheight 300 –matrix matrices\edna.mat –lower 33 –upper 67. Comparisons between species used masked sequences. Unmasked sequences were used in the comparison of mouse 5’ and 3’ flanks.

Figures of the probability of conservation were generated using ggplot2 [51].

## Additional files

Additional file 1:
***Sry*** **coding sequences.** (TXT 12 kb) Additional file 2:
***Sry*** **5’ flanking sequences.** Repeat-masked 10 kb sequences from 5’ of*Sry.* (TXT 149 kb) Additional file 3:
***Sry*** **3’ flanking sequences.** Repeat-masked 10 kb sequences from 3’ of *Sry.* (TXT 146 kb) Additional file 4:
**Sequence of the SPCI.** The sequence of the SPCI from human and mouse. (TXT 250 bytes) Additional file 5:
**Additional tables.** Tables required to reproduce results, but not necessary to understand the paper. (PDF 245 kb) Additional file 6:
**CentriMo input sequences.** The 600 bp 5’ flanking sequences used in the CentriMo analysis. (TXT 10 kb) Additional file 7:
**Full CentriMo results.** A single interactive HTML file containing all results from the CentriMo analysis. (HTML 795 kb) Additional file 8:
**Processed CentriMo results.** Significant results from the CentriMo analysis in an alternative format. (XLS 70 kb) Additional file 9:
**Windowed FIMO results.** Processed results of FIMO analysis with 1 kb windows. (XLS 39 kb) Additional file 10:
**Windowed FIMO results.** Processed results of FIMO analysis with 10 kb windows. (XLS 81 kb)
