# Supplementary material for: Conservation analysis of sequences flanking the testis-determining gene Sry in 17 mammalian species
Source: BMC Dev Biol. 2015 Oct 6;15:34. doi: 10.1186/s12861-015-0085-6 (PMC4595323; doi:10.1186/s12861-015-0085-6)
Supplement: Additional file 5: — Additional tables. Tables required to reproduce results, but not necessary to understand the paper. (PDF 245 kb) [file 12861_2015_85_MOESM5_ESM.pdf]

Supplementary Table 1 RepeatMasker parameter used for masking each sequence

Species	Value selected for DNA source when using RepeatMasker
Antelope	Mammal (other than below)
Bat	Mammal (other than below)
Chimpanzee	Mammal (other than below)
Cow	Cow
Ferret	Mammal (other than below)
Goat	Mammal (other than below)
Horse	Mammal (other than below)
Human	Human
Macaque	Mammal (other than below)
Marmoset	Mammal (other than below)
Mouse	Mouse
Pig	Pig
Rabbit	Mammal (other than below)
Sheep	Mammal (other than below)
Tiger	Mammal (other than below)
Walrus	Mammal (other than below)
Whale	Artiodactyls and whales

Where sequences used in this study were repeat-masked, the values above were used for RepeatMasker's DNA source parameter.

Supplementary Table 2 Positions relative to XSS used to align ungapped 5' flanking sequences.

Species	Position
Antelope	-62
Bat	-172
Chimpanzee	-130
Cow	-145
Ferret	-36
Goat	-62
Horse	-148
Human	-130
Macaque	-130
Marmoset	-130
Mouse	-327
Pig	-127
Rabbit	-108
Sheep	-65
Tiger	-156
Walrus	-127
Whale	-154

Positions listed were used to align ungapped sequences on a proximal SP-like motif in preparation for analysis with CentriMo.
